# Elevated Mitochondrial Reactive Oxygen Species and Cellular Redox Imbalance in Human NADPH-Oxidase-Deficient Phagocytes

**DOI:** 10.3389/fimmu.2017.01828

**Published:** 2017-12-21

**Authors:** Martina Sundqvist, Karin Christenson, Halla Björnsdottir, Veronica Osla, Anna Karlsson, Claes Dahlgren, David P. Speert, Anders Fasth, Kelly L. Brown, Johan Bylund

**Affiliations:** ^1^The Phagocyte Research Group, Department of Rheumatology and Inflammation Research, University of Gothenburg, Gothenburg, Sweden; ^2^Department of Pediatrics, Centre for Understanding and Preventing Infection in Children, University of British Columbia, Vancouver, BC, Canada; ^3^Department of Pediatrics, University of Gothenburg, Gothenburg, Sweden; ^4^Department of Pediatrics, The University of British Columbia at The British Columbia Children’s Hospital Research Institute (Formerly the Child and Family Research Institute), Vancouver, BC, Canada; ^5^Department of Oral Microbiology and Immunology, Sahlgrenska Academy at the University of Gothenburg, Gothenburg, Sweden

**Keywords:** chronic granulomatous disease, oxidative stress, reactive oxygen species, inflammation, cytokine

## Abstract

Chronic granulomatous disease (CGD) is caused by mutations in genes that encode the NADPH-oxidase and result in a failure of phagocytic cells to produce reactive oxygen species (ROS) *via* this enzyme system. Patients with CGD are highly susceptible to infections and often suffer from inflammatory disorders; the latter occurs in the absence of infection and correlates with the spontaneous production of inflammatory cytokines. This clinical feature suggests that NADPH-oxidase-derived ROS are not required for, or may even suppress, inflammatory processes. Experimental evidence, however, implies that ROS are in fact required for inflammatory cytokine production. By using a myeloid cell line devoid of a functional NADPH-oxidase and primary CGD cells, we analyzed intracellular oxidants, signs of oxidative stress, and inflammatory cytokine production. Herein, we demonstrate that phagocytes lacking a functional NADPH-oxidase, namely primary CGD phagocytes and a gp91^phox^-deficient cell line, display elevated levels of ROS derived from mitochondria. Accordingly, these cells, despite lacking the major source of cellular ROS, display clear signs of oxidative stress, including an induced expression of antioxidants and altered oxidation of cell surface thiols. These observed changes in redox state were not due to abnormalities in mitochondrial mass or membrane integrity. Finally, we demonstrate that increased mitochondrial ROS enhanced phosphorylation of ERK1/2, and induced production of IL8, findings that correlate with previous observations of increased MAPK activation and inflammatory cytokine production in CGD cells. Our data show that elevated baseline levels of mitochondria-derived oxidants lead to the counter-intuitive observation that CGD phagocytes are under oxidative stress and have enhanced MAPK signaling, which may contribute to the elevated basal production of inflammatory cytokines and the sterile inflammatory manifestations in CGD.

## Introduction

In response to exogenous stimuli and endogenous danger signals, phagocytes assemble the NADPH-oxidase to generate vast amounts of highly reactive oxidants, called reactive oxygen species (ROS) (1, 2). This stimulus-induced, rapid production of NADPH-oxidase-derived ROS (herein called phoxROS) is commonly referred to as the phagocytic oxidative burst and is imperative for the oxidative killing of microbes. Chronic granulomatous disease (CGD) is a rare genetic disorder in which mutations in genes encoding the NADPH-oxidase compromise the ability of phagocytes to generate phoxROS. Not surprising, CGD patients are highly susceptible to particular strains of fungi and bacteria that are resistant to non-oxidative killing methods of immune cells (3–5). CGD patients also frequently suffer from severe and debilitating inflammatory conditions (5). This aspect of the disease is not well understood but correlates with enhanced basal and stimulus-induced inflammatory signaling and pro-inflammatory cytokine production by CGD immune cells (6–10).

While the NADPH-oxidase is the primary source of oxidants in phagocytes, oxidants are also produced by other oxidases and peroxidases in subcellular compartments (2, 11). For non-phagocytic cells, one major source of cellular oxidants is the mitochondrial electron transport chain (12). Regardless of source, all oxidants and their counterpart antioxidants engage in redox (reduction-oxidation) reactions in the cell. Redox reactions are integral to an array of cellular processes, from homeostatic cellular respiration to signal transduction and oxidative killing of microbes. It has also been reported that oxidants are required for the assembly of NLRP3-containing inflammasomes (13, 14) and subsequent production of pro-inflammatory cytokines in response to endogenous and foreign stimuli (15, 16). Precisely which oxidants that promote TLR-induced inflammatory signaling is difficult to ascertain and investigations of this nature often produce conflicting results. Although ROS produced by mitochondria (herein called mtROS) are simply byproducts of cellular respiration (17, 18), there is evidence that mtROS promote TLR-induced inflammatory cytokine production (19–21). It has also been reported that phoxROS produced in response to TLR agonists promote inflammation (22). In both cases, mechanistic evidence for a direct link between TLR-activation, ROS production, and cell-signaling is lacking. Nonetheless, observations that ROS promote inflammation are contrary to the *in vitro* and *in vivo* evidence that for CGD patients and cells, the absence of phoxROS correlates with heightened inflammation (6–10).

In this study, we wanted to address this apparent paradox that ROS drive inflammatory signaling yet CGD is a hyper-inflammatory condition. We used a human cell line and primary human CGD phagocytes, both of which are naturally devoid of phoxROS. Like phagocytes obtained from individuals with CGD, the phoxROS-deficient cell line produced higher levels of basal inflammatory cytokines (IL1β, TNFα, and IL8) compared to the control (ROS-competent) cell line. Moreover, both phoxROS-deficient primary and cell line phagocytes displayed increased levels of intracellular ROS derived from mitochondria (mtROS). Elevated mtROS was constitutive, not requiring cellular activation, and was not attributable to alterations in mitochondrial mass or membrane integrity. The increase in mtROS levels was sufficiently high to induce antioxidant expression, a change in protein thiol oxidation, rapid phosphorylation of ERK1/2, and elevated production of pro-inflammatory cytokines (IL8). Thus, we provide evidence that phagocytes lacking the phoxROS-generating NADPH-oxidase are under oxidative stress due to increased levels of ROS from mitochondria which results in increased inflammatory MAPK signaling and cytokine production. These data suggest that phoxROS-deficient immune cells actively contribute to the chronic, debilitating inflammatory disorders that spontaneously develop in individuals with CGD.

## Materials and Methods

### Reagents

RPMI 1640 were from PAA Laboratories GMbH and fetal calf serum (FCS), l-glutamine, sodium pyruvate, penicillin, streptomycin, Cell Dissociation Reagent (GIBCO) and Restore Western blot stripping buffer were purchased from Thermo Fisher Scientific GTF AB (Gothenburg, Sweden), the High-Capacity cDNA Reverse Transcription kit were from Applied Biosystems, Thermo Fisher Scientific (Mississauga, ON, Canada) and human IL8 ELISA (BMS204/3) from eBioscience, Thermo Fisher Scientific (Mississauga, ON, Canada). Dimethyl sulfoxide (DMSO), PMA, Antimycin A, and bovine serum albumin (BSA) were all purchased from Sigma-Aldrich (St. Louis, MO, USA). Dextran was from Pharmacosmos [Holbaek, Denmark and horseradish peroxidase (HRP)] and catalase were from Boehringer Mannheim (Mannheim, Germany). MitoSOX™ Red, 2′,7′-Dichlorofluorescin diacetate (DCFDA), Mitotracker Green FM, Alexa Fluor^®^ 633 C_5_ maleimide (ALM-633), Amplex Red, *E. coli* RNase and SuperScript™ III Platinum^®^ Two-Step qRT-PCR Kit with SYBR^®^ Green were all from Molecular Probes/Invitrogen (Grand Island, NY, USA). Tetramethylrhodamine (TMRE), carbonyl cyanide 4-(trifluoromethoxy) phenylhydrazone (FCCP) were purchased from Abcam (Cambridge, UK), Pefabloc protease inhibitor cocktail were from Roche Diagnostic (Mannheim, Germany) and HALT protease inhibitor cocktail from Pierce, Thermo Fisher Scientific (Mississauga, ON, Canada). The cytokine bead array assay (CBA) and skimmed milk were from Becton Dickinson Biosciences (San Jose, CA, USA) and the RNeasy Mini kit were from Qiagen (Hilden, Germany). Rabbit anti-phospho-ERK1/2 and rabbit anti-ERK1/2 antibodies were from Cell Signalling Technology (MA, USA) and HRP-conjugated anti-rabbit antibody from DAKO (Stockholm, Sweden). The clarity western ECL substrate was from Bio-Rad (Solna, Sweden) and the Triton X-100 was from Merck (Darmstadt, Germany).

### Cell Lines

The human myelomonoblastic cell line PLB-985 (PLB) (23) and gp91^phox^-deficient clone (hereafter referred to as X-PLB cells) (24) were gifts from Dr. M. Dinauer. Non-differentiated PLB cells produce little or no phoxROS, but when differentiated into phagocytes (neutrophils or monocytes) according to established protocols the PLB, but not the X-PLB, are capable of producing a significant “burst” of phoxROS (6, 23, 24). In brief, PLB and X-PLB cells were cultured in RPMI 1640 containing 10% FCS, 2 mM l-glutamine, 1 mM sodium pyruvate, 100 U/mL penicillin and 100 µg/mL streptomycin (complete RPMI) at 37°C, 5% CO_2_. Differentiation into neutrophil-like cells was achieved by addition of 1% DMSO for 5 days (23) while differentiation into monocyte-like cells was done with 50 nM PMA for 15–18 h. After maturation, cells were washed and allowed to rest for 1–2 h in DMSO/PMA-free complete RPMI at 37°C, 5% CO_2_ (25). Adherent monocytes were detached from plates by incubation with Versene’s solution for 15 min or with Cell Dissociation Reagent according to manufacturer’s instructions.

### Primary Cells

Peripheral blood was diluted 1:1 with 2% dextran to sediment erythrocytes after which PMN were isolated on a Ficoll gradient, washed and kept on ice in Krebs–Ringer phosphate buffer (KRG, pH 7.3) with Ca^2+^ (1 mM) as previously described (25). Due to limited sample (blood volume) from patient #1, whole leukocyte preparations (remaining after erythrocyte lysis) were used in analysis of this patient and its corresponding control. All blood samples from healthy controls and CGD patients contained leukocyte counts within the normal range.

### Flow Cytometry Analysis

Unless stated otherwise, a minimum of 10,000 gated events were collected on a BD Biosciences Accuri C6 or a FACS Canto flow cytometer (Becton Dickinson, San Jose, CA, USA). Primary leukocyte populations (neutrophils and monocytes) were distinguished by gating on forward scatter (FSC, size) versus side scatter (SSC, density). All collected data were analyzed with FlowJo software (TreeStar Inc., OR, USA).

### ROS Production

To induce phoxROS production, cell lines and primary cells were exposed to PMA (50 nM) and phoxROS were detected using an isoluminol-enhanced chemiluminescence (CL) system (26) with a Mithras LB940 or a Biolumat LB 9505 (Berthold technologies, Bad Wildbad, Germany). The reaction mixture (200 µL) contained 0.1–1 × 10^6^ cells, 4 U/mL HRP, 2 × 10^−5^ M isoluminol in KRG supplemented with Ca^2+^ (1 mM) and PMA (50 nM) as the stimulus. For total intracellular ROS, cell lines (5 × 10^5^ cells) were stained for 30 min at 37°C with 10 µM DCFDA in the absence and presence of PMA (50 nM; last 15 min of staining) then washed and analyzed by flow cytometry (26). Mitochondrial-derived ROS (mtROS) were detected in cells (2.5–5 × 10^5^) stained at 37°C for 20–30 min with 5 µM (cell lines) or 10 µM (primary cells) MitoSOX™ Red. After staining, the cells were washed and analyzed by flow cytometry. As a precaution, cells were analyzed within 10–20 min of the completion of staining as MitoSOX tends to accumulate in the nucleus after approximately 40 min [(27) and our unpublished observations]. To increase mtROS production, cell lines were incubated with Antimycin A (20 µM) during the last 15 min of the MitoSOX staining.

### Mitochondrial Mass and Membrane Integrity

For studies of mitochondrial mass and integrity we used two different assays. Mitotracker Green FM is a mitochondria-specific probe and indicator of mitochondrial mass, targeting these organelles independent of mtROS levels and mitochondrial membrane potential. For measurements of mitochondrial membrane potential TMRE staining was used with or without treatment with FCCP, which depolarizes mitochondrial membranes. Cell lines (5 × 10^5^ cells) were stained at 37°C for 20–30 min with 100 nM Mitotracker Green FM or 400 nM TMRE with or without pre-treatment for 10 min with 20 µM FCCP.

### Cell Surface Thiols

Reduced free thiol groups (SH-) were detected by staining neutrophil-differentiated cells (5 × 10^5^ cells) and monocyte-differentiated cells (2.5 × 10^5^ cells) from PLB and X-PLB cell lines with 5 µM Alexa Fluor^®^ 633 C_5_ maleimide (ALM-633) for 15 min on ice in the dark. Cells were then washed and analyzed by flow cytometry.

### Cellular Reducing Capacity

PLB and X-PLB cell lines were lysed at 2 × 10^6^ cells/mL with Triton X-100 (0.1%) in the presence of Pefabloc protease inhibitor cocktail (1 mM). Lysates were vortexed and centrifuged at 4°C for 25 min, after which supernatants were stored at −80°C. For analysis of H_2_O_2_-reducing capacity, cell lysates were diluted 1:100 in PBS and incubated with H_2_O_2_ (10 µM) at 37°C for 15 min. Remaining H_2_O_2_ in the samples was detected with Amplex Red (50 µM) in the presence of HRP (1 U/mL) using a Mithras LB940 dual-function fluorometer-luminometer (Berthold Technologies, Bad Wildbad, Germany). As a positive and negative control, respectively, catalase (2,000 U/mL; that catalyzes the transfer of H_2_O_2_ to H_2_O) or buffer was used. Data was calculated by (1 − ((sample − positive control)/(negative control − positive control)) × 100), expressed as % of control.

### Cytokine Detection

Monocyte-differentiated cell lines (1 × 10^6^ cells/mL) were incubated in PMA-free complete RPMI at 37°C, 5% CO_2_ for 24 h after which tissue culture supernatants were centrifuged, collected, and stored at −80°C prior to analysis of TNFα, IL1β, and IL8 by CBA or ELISA. Following incubation in the absence and presence of Antimycin A (20 µM), 5 × 10^5^–1 × 10^6^ monocyte-differentiated cells were lysed at 2.5 × 10^6^ cells/mL in lysis buffer (10 mM Tris pH 7.5, 150 mM NaCl, 2 mM EDTA, 1% Triton X-100) containing HALT protease inhibitor cocktail for 10 min on ice. Lysates were vortexed and centrifuged at 4°C for 25 min, supernatants were collected and stored at −80°C prior to analysis of IL8 by standard sandwich ELISA according to manufacturer’s protocols.

### Quantitative Real-time PCR

Total RNA was isolated from monocyte-differentiated PLB and X-PLB cells using RNeasy Mini kit. The cDNA was prepared from 75 ng of RNA using High-Capacity cDNA Reverse Transcription kit. Remaining RNA was digested at 37°C for 30 min with *E. coli* RNase, after which one-tenth of the RT reaction mixture was used to evaluate the expression of mitochondrial-specific superoxide dismutase (mnSOD/SOD2, Forward primer: GAC CTG CCC TAC GAC TAC GG, Reverse Primer: TTC AGG TTG TTC ACG TAG GCC) and cytosolic thioredoxin (TXN/TRX1, Forward primer: TGA AGC AGA TCG AGA GCA AGA C, Reverse Primer: TTC ATT AAT GGT GGC TTC AAG C) relative to the expression of β2-microglobulin (Forward primer: CTC GCG CTA CTC TCT CTC TTT CT, Reverse primer TGC TCC ACT TTT TCA ATT CTC T) using the SuperScript™ III Platinum^®^ Two-Step qRT-PCR Kit with SYBR^®^ Green.

### ERK1/2 Immunoblotting

PMN from healthy control buffycoats were suspended (1 × 10^7^ PMN/mL) in KRG and treated with Antimycin A (20 µM), buffer (KRG) or IL1β (100 ng/mL) at 37°C. After 1–20 min, samples were lysed on ice (1 mM Pefablock, 0.1% Triton X 100) and centrifuged twice at 4°C, 16,100 × *g*. Lysates were mixed 1:5 with reducing sample buffer, boiled for 5 min and resolved by SDS-PAGE. Proteins were transferred to nitrocellulose membranes, blocked with PBS containing skimmed milk (5%)/Tween20 (0.05%) at RT for 30 min or overnight at 4°C. The membranes were then washed (PBS/0.05% Tween20) and incubated with antibodies (all diluted in PBS/0.05% Tween20/5% BSA). Membranes were incubated with rabbit anti-phospho ERK1/2 antibody (diluted 1/200, 2 h at RT or overnight at 4°C) followed by HRP-conjugated anti-rabbit antibody (diluted 1/2,000, 1 h at RT or overnight at 4°C), washed and developed with clarity western ECL substrate according to manufacturer’s instructions. Luminescence was analyzed in a Molecular Imager ChemiDoc XRS by Quantity One Software (Bio-Rad Laboratories). Membranes were washed and stripped (15 min at RT) with Restore Western blot stripping buffer then incubated with rabbit anti-ERK1/2 antibody (diluted 1/1,000 for 2 h at RT or overnight at 4°C) followed by HRP-conjugated anti-rabbit antibody and developed as previously described.

### Statistical Analysis

Statistical calculations were performed in GraphPad Prism software version 6.0a (Graphpad Software, San Diego, CA, USA). The PLB and X-PLB phagocytes that were matured and analyzed on the same date, as well as treated on the same day, and control and CGD blood phagocytes that were sampled and analyzed on the same date were regarded as paired data and evaluated by paired Student’s *t*-tests or repeated measurement one-way ANOVA followed by Sidak’s multiple comparison test. The data in Figure 2A were obtained from competitive cDNA microarray technology; data available at https://www.ebi.ac.uk/arrayexpress/experiments/E-FPMI-9/. On each microarray slide, the RNA from patient phagocytes competed with the RNA from control phagocytes for binding to the probes. Raw data are inherently a relative ratio of gene expression in the CGD cells to that in the control cells (with expression in the control sample = 1) with statistical significance determined by Arraypipe software (in-house) as previously described (6). *p*-Values equal to or less than 0.05 were regarded significant and are indicated by (*) *p* < 0.05, (**) *p* < 0.01, or (***) *p* < 0.001.

## Results

### phoxROS-Deficient PLB Phagocytes Have Constitutively Elevated Cytokines and Intracellular ROS

**Figure 1:**
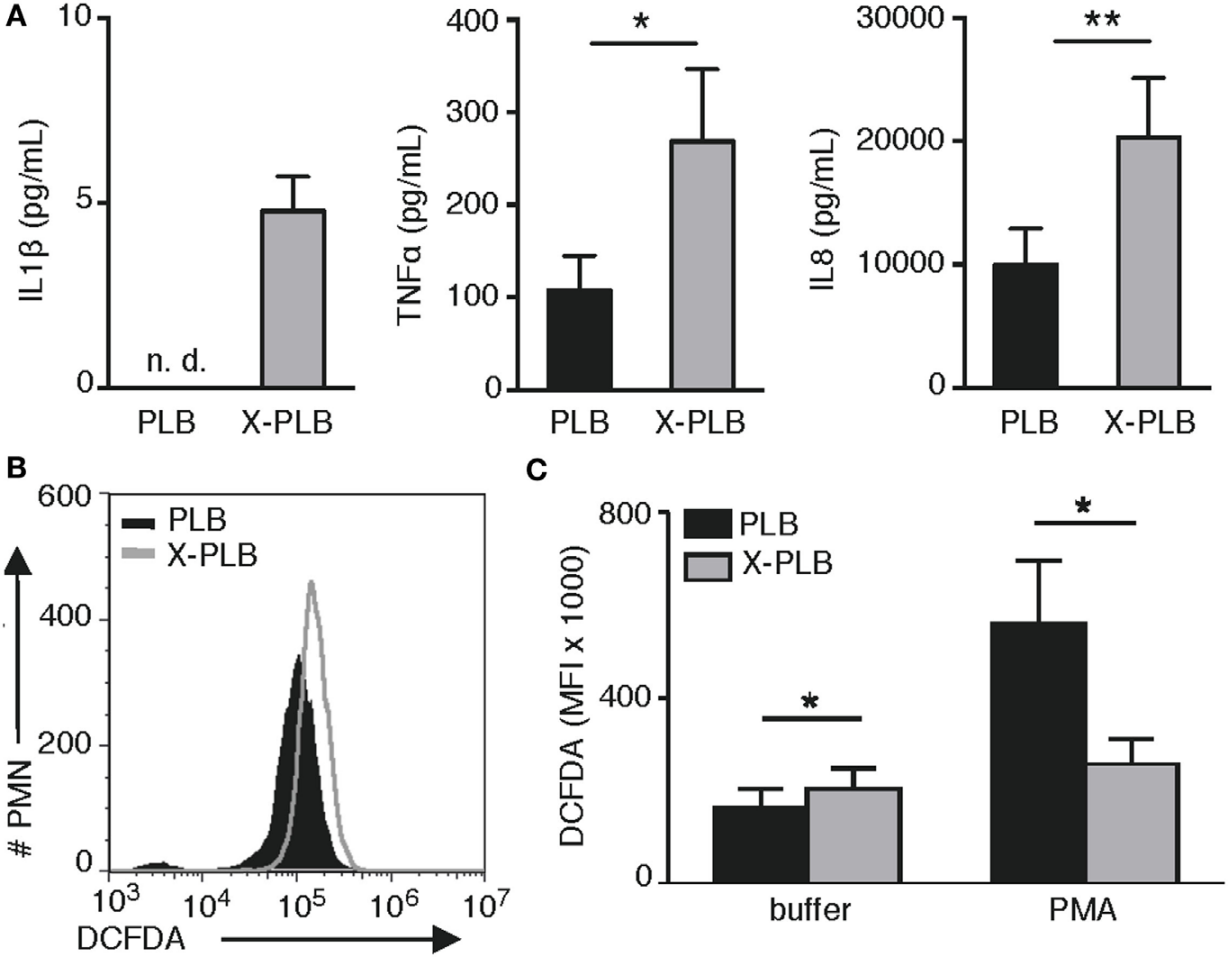
Cytokine production and intracellular (ROS) in PLB and phoxROS-deficient X-PLB phagocytes. **(A)** IL1β, TNFα, and IL8 in tissue culture supernatants from non-activated PLB (black) and X-PLB (gray) monocytes measured by CBA assay and ELISA (shown as mean + SEM of three to nine independent experiments). IL1β production by PLB monocytes was below the lower limit of detection (not detectable, n.d.). **(B)** A representative flow cytometry histogram of (DCFDA) staining and **(C)** the average (MFI) + SEM of DCFDA staining (*n* = 4) in non-activated PLB (black) and X-PLB (gray) PMN in the absence (buffer) and presence of phoxROS stimulant, (PMA).

In this study, we investigated the role of ROS in inflammatory signaling (14, 15) specifically in the context of heightened production of cytokines by CGD cells (5–9) that, due to a dysfunctional NADPH-oxidase, are devoid of the primary source of cellular ROS. We utilized a human myeloid cell line [PLB (23)] together with its genetically modified CGD variant that is deficient in phoxROS [X-PLB (24)]. Upon differentiation, the PLB cells express components of the NADPH-oxidase and can produce phoxROS while the X-PLB cells are unable to do so. First, we measured the basal production of inflammatory cytokines by the PLB and X-PLB phagocytes and verified that, like primary murine and human CGD cells, phoxROS-deficient X-PLB cells spontaneously produced higher concentrations of the pro-inflammatory cytokines IL1β, TNFα, and IL8 as compared to the phoxROS-competent PLB cells (Figure 1A) (6, 23).

Next, we assessed baseline and stimulated levels of intracellular ROS in the PLB and X-PLB cells using DCFDA, a generic stain for intracellular oxidants. Examples of elevated oxidant levels in primary CGD cells in the absence of stimulation and using this stain can be found in the literature [for examples, see Ref. (28, 29)]. Similarly, our data show that resting X-PLB phagocytes displayed higher baseline DCFDA fluorescence as compared to resting PLB phagocytes (Figures 1B,C). As a control, PLB and X-PLB cells were stained with DCFDA and exposed to PMA, a potent activator of the NADPH-oxidase. As expected, PMA caused an increase in DCFDA fluorescence in PLB phagocytes (DCFDA reacting with induced phoxROS), but not X-PLB cells (Figure 1C). Thus, like primary human CGD cells, unstimulated, phoxROS-deficient X-PLB phagocytes produce higher levels of constitutive intracellular ROS and produce higher baseline levels of inflammatory cytokines as compared to control (PLB) cells.

### phoxROS-Deficient Phagocytes Display Signs of Oxidative Stress

As the observed increase in intracellular ROS in the phoxROS-deficient cells was relatively modest, we next looked for supporting evidence for an overabundance of intracellular oxidants in these cells by analyzing common signs of oxidative stress, including the induced expression of antioxidants, extent of protein thiol oxidation, and overall antioxidative capacity of the cell.

**Figure 2:**
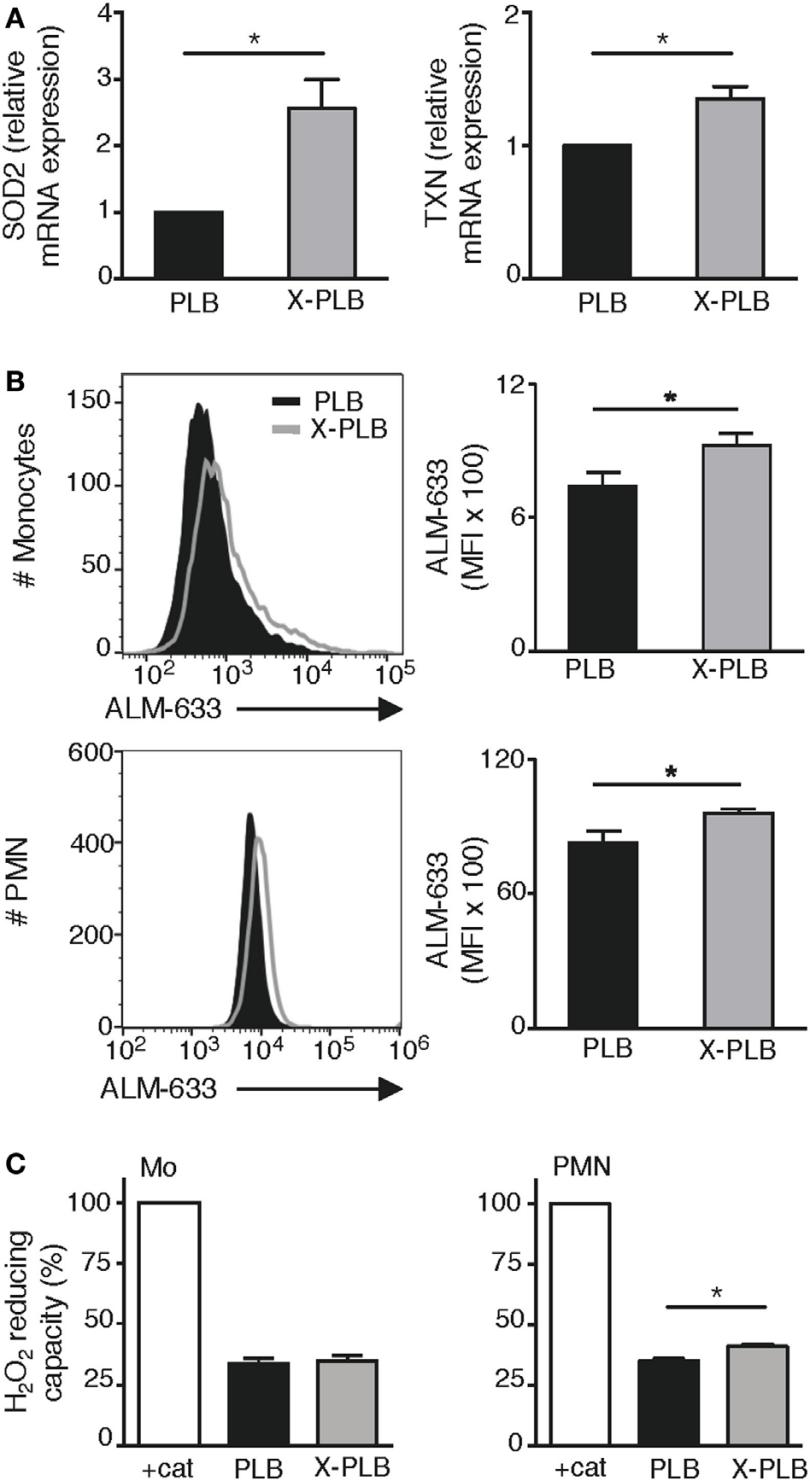
Antioxidant response in phoxROS-deficient phagocytes. **(A)** Relative expression of (SOD2) (left) and *TXN* (right) mRNA (normalized to β2-microglobulin expression) in PLB (black) and X-PLB (gray) monocytes (*n* = 3). **(B)** Levels of reduced thiols on PLB (black) and X-PLB (gray) monocytes (top, *n* = 5) and PMN (bottom, *n* = 5), left panels show representative flow cytometry histogram and right panels (bar graphs) show average (MFI) + SEM of ALM-633 staining. **(C)** The H_2_O_2_-reducing capacity (mean + SEM) in lysates derived from PLB (black) and X-PLB (gray) monocytes (left panel, *n* = 3), and PMN (right panel, *n* = 4) as measured by Amplex Red fluorescence of remaining H_2_O_2_ in relation to buffer in the presence of catalase (100% control).

We have previously performed microarray analysis of basal gene expression in blood monocytes obtained from four patients with X-linked CGD (6). Within that dataset, we specifically looked at the expression of antioxidants and found that the basal expression of superoxide dismutase-2 (*SOD2*) and Thioredoxin (*TXN*) were significantly elevated in CGD monocytes compared to control monocytes (Table 1; 1.9-fold more *SOD2* mRNA, *p* = 0.008, and 1.4-fold more *TXN* mRNA, *p* = 0.04, in CGD monocytes compared to controls). The elevated expression of *SOD2* and *TXN* was sustained even after 24 h in culture (1.9-fold more *SOD2* mRNA, *p* = 0.0005, and 1.5-fold more *TXN* mRNA, *p* = 0.076). Expression of these antioxidant genes was subsequently tested in the cell lines. Results show that the mRNA expression of *SOD2* and *TXN* was elevated in X-PLB monocytes, compared to PLB monocytes (Figure 2A). The differences in expression between the X-PLB monocytes and PLB monocytes (*SOD2*; 2.56 ± 0.44, *n* = 3 and *TXN*; 1.35 ± 0.09, *n* = 3, fold higher expression in X-PLB monocytes) were strikingly similar in magnitude to those observed in our microarray analysis of differential gene expression between the primary CGD and control monocytes.

**Table 1:** Relative basal expression of *SOD2* and *TXN* in chronic granulomatous disease (CGD) monocytes compared to monocytes from healthy individuals.

Gene	4 h		24 h	
	Fold increase^a^	*p*-Value	Fold increase^a^	*p*-Value
*SOD2*	*1.9*	0.0079	1.9	0.0005
*TXN*	*1.4*	0.0417	1.5	0.0758

*^a^Increased mRNA expression in four individuals with gp91^phox^-deficient CGD relative to five healthy individuals following incubation for 4- and 24-h *ex vivo* in the absence of stimulation. Data are from a previously published dataset (6)*.

Protein thiol groups are highly sensitive to redox reactions, oscillating between a reduced free thiol group (-SH) and an oxidized disulfide bond (S-S). A change in the -SH: S-S ratio can thus be an indicator of redox imbalance and cellular oxidative stress (30). We evaluated the extent of cell surface protein thiol reduction on PLB and X-PLB phagocytes with the fluorescent probe ALM-633 and found a significantly elevated ALM-633 staining on X-PLB phagocytes compared to PLB phagocytes (Figure 2B). Thus, protein thiols are reduced to a higher extent on X-PLB phagocytes as compared to PLB phagocytes. Finally, we measured the antioxidative capacity of cell lysates and observed that X-PLB phagocytes have similar (for monocytes) or marginally greater (for neutrophils) H_2_O_2_-reducing capacity than PLB phagocytes (Figure 2C).

Together, these data demonstrate that phoxROS-deficient primary phagocytes (Table 1) and X-PLB cells (Figures 2A–C) show clear signs of redox imbalance, as evidenced by the increased expression of antioxidant genes (*SOD2* and *TXN*) and reduced surface thiols. The results support our observation that intracellular oxidants (Figures 1B,C) are elevated in these cells to an extent that warrants a cellular response.

### phoxROS-Deficient X-PLB Cells Have Elevated Levels of Mitochondria-Derived ROS

The antioxidant SOD2 is a mitochondria-restricted enzyme that is solely induced when levels of ROS in the mitochondria fail to be neutralized (31, 32). To determine if the elevated levels of intracellular ROS in resting CGD phagocytes represent an abundance of mitochondrial ROS (mtROS), we employed the fluorescent dye MitoSOX that specifically detects ROS generated as a by-product of mitochondrial respiration. We first verified by confocal microscopy that the MitoSOX stain was localized to mitochondrial structures, and observed an extensive network of staining for both PLB (Figure S1A in Supplementary Material) and X-PLB phagocytes (not shown).

**Figure 3:**
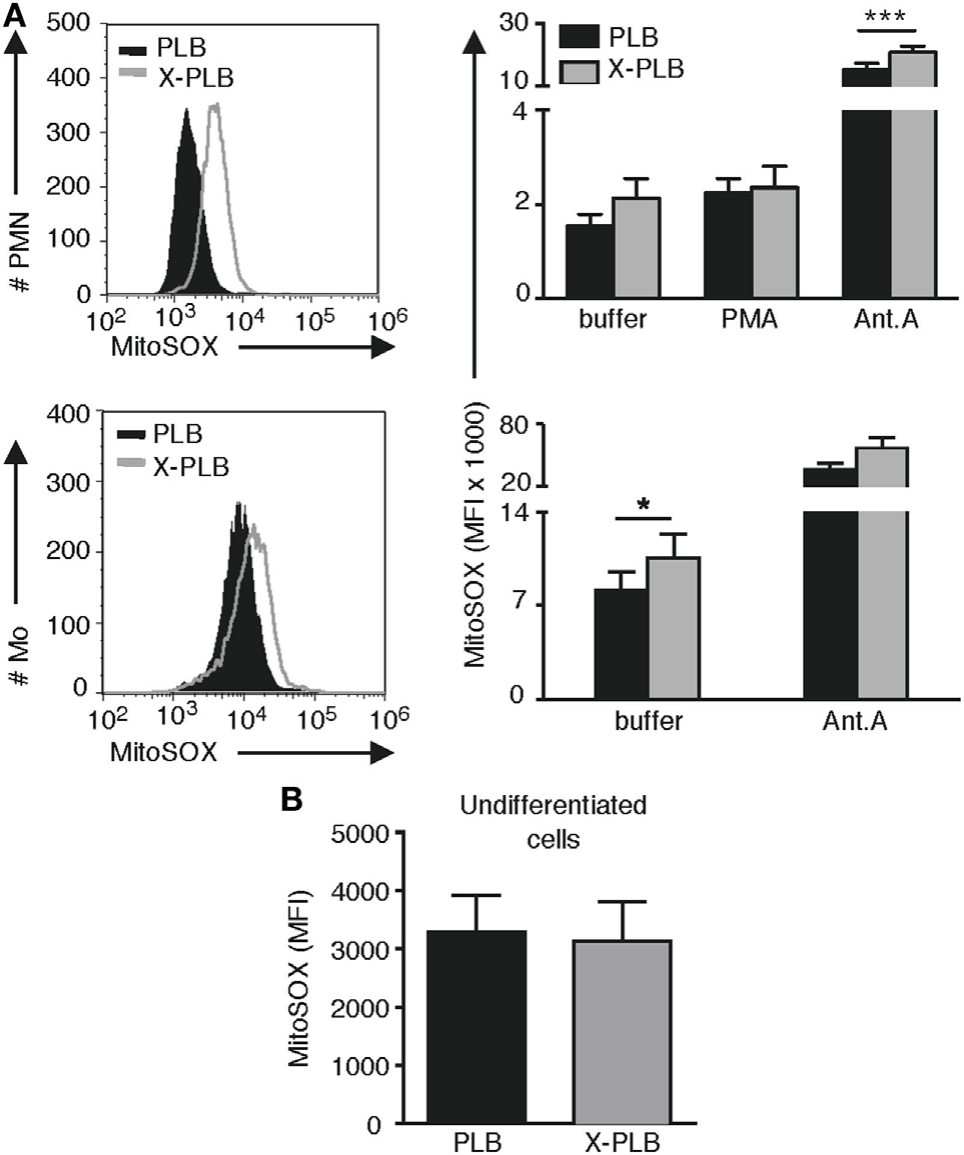
(mtROS) in control and phoxROS-deficient phagocytes. **(A)** Representative flow cytometry histograms (left panels) of MitoSOX staining and average (MFI) + SEM of MitoSOX staining (bar graphs, right panels) in non-activated PLB (black) and X-PLB (gray) PMN (*n* = 9) and monocytes (*n* = 3) in the absence (buffer) or presence of potent stimuli of phoxROS [(PMA); *n* = 8] or mtROS (Antimycin A; PMN, *n* = 6; Mo, *n* = 3). **(B)** Mean MFI + SEM of MitoSOX staining of non-activated, undifferentiated PLB (black) and X-PLB (gray) cells (*n* = 6).

To quantify the intensity of MitoSOX staining, PLB and X-PLB cells were analyzed by flow cytometry. Results demonstrate that X-PLB monocytes had significantly elevated levels of mtROS (higher intensity MitoSOX fluorescence) compared to PLB monocytes in the resting state (Figure 3A). Antimycin A is a mitochondrial electron transport chain inhibitor that potently induces mtROS in both PLB and X-PLB phagocytes (Figure 3A), but that does not influence phoxROS production (shown in Figure S2 in Supplementary Material). Interestingly, when mtROS was measured in the cell lines *prior to* differentiation into phagocytes (i.e., prior to expression of the NADPH-oxidase in PLB cells), there was no difference in mtROS (Figure 3B), indicating that the difference in mtROS seen after differentiation is indeed coupled to the expression of a functional NADPH-oxidase.

### PLB and phoxROS-Deficient X-PLB Phagocytes Have Comparable Mitochondrial Mass and Membrane Integrity despite Differences in mtROS Production

**Figure 4:**
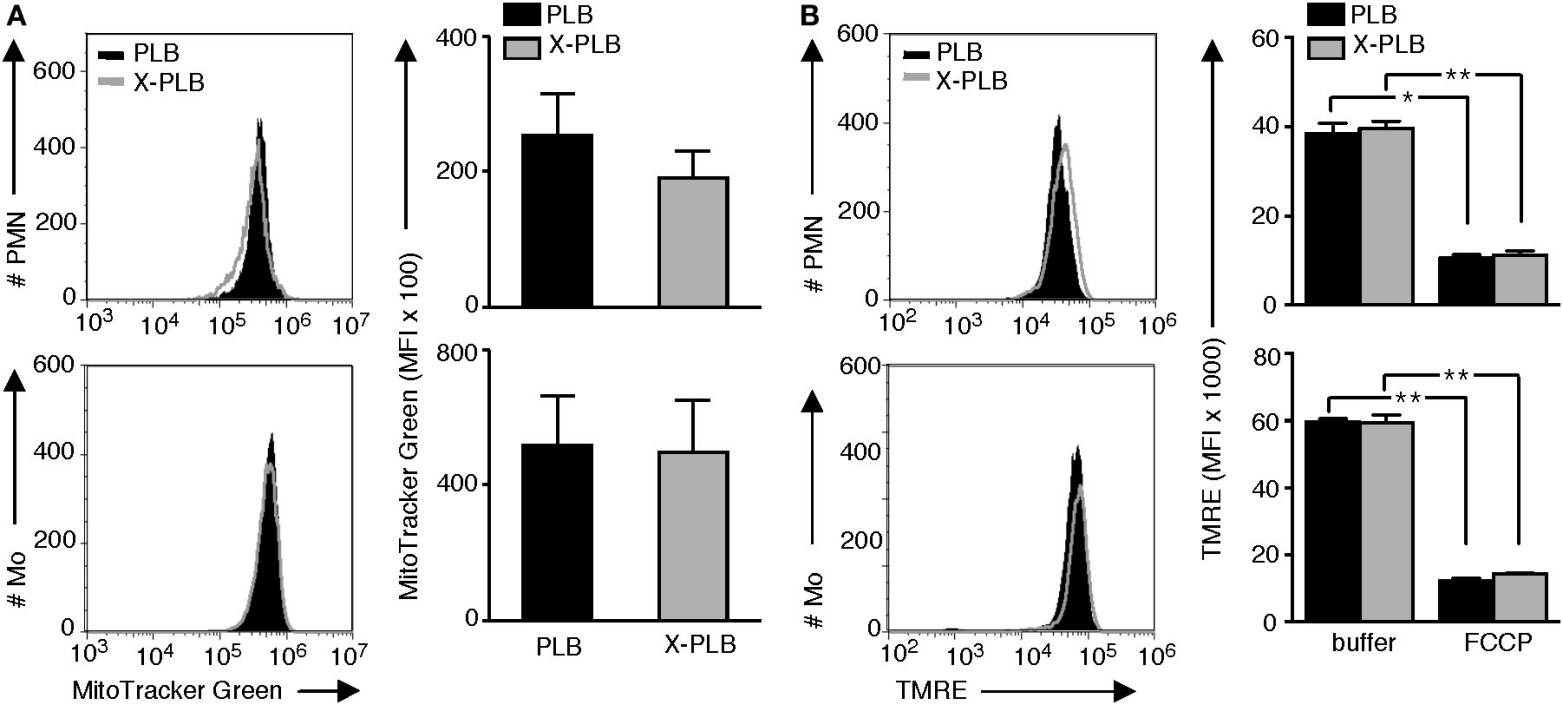
Mitochondrial mass and membrane integrity in PLB and phoxROS-deficient X-PLB phagocytes. **(A)** Representative flow cytometry histograms (left panels) and average (MFI) + SEM of MitoTracker Green staining (right panels, bar graphs) in PLB (black) and X-PLB (gray) PMN (upper panels, *n* = 5) and monocytes (lower panel, *n* = 7). **(B)** Representative flow cytometry histograms (left panels) and average MFI + SEM of (TMRE) staining (right panels, bar graphs) in PLB (black) and X-PLB (gray) PMN (upper panel, *n* = 3) and monocytes (lower panel, *n* = 3) pre-incubated in the absence (−) or presence of FCCP, which depolarizes mitochondrial membranes.

To rule out the possibility that the observed difference in MitoSOX fluorescence was due to differences in available binding sites (i.e., mitochondria) for this probe, we evaluated the mitochondrial mass in the PLB and X-PLB phagocytes. Using MitoTracker Green, a mitochondrial targeting probe that is widely used for visualizing and quantifying these organelles, a network of mitochondria similar to that observed with MitoSOX (Figure S1A in Supplementary Material) was illuminated (Figure S1B in Supplementary Material). Quantification of MitoTracker Green staining by flow cytometry analysis yielded similar results with no statistically significant differences for PLB and X-PLB phagocytes (Figure 4A), indicating that they have comparable mitochondrial mass.

Depolarization of mitochondrial membranes can be associated with increased production of mtROS. To evaluate mitochondrial membrane potential PLB and X-PLB phagocytes were incubated with TMRE, a dye that accumulates in polarized mitochondria. We first confirmed that cells with depolarized mitochondrial membranes, achieved by treating primary neutrophils from healthy controls with FCCP, had detectably decreased TMRE fluorescence (Figure S3A in Supplementary Material) and a correspondingly elevated MitoSOX fluorescence (Figure S3B in Supplementary Material). Despite the difference in MitoSOX staining between PLB and X-PLB phagocytes, TMRE staining intensity was never lower in X-PLB phagocytes (Figure 4B) as would be expected if membrane depolarization was responsible for the observed increase in mtROS. Thus, the increased mtROS in X-PLB phagocytes was not a result of increased mitochondrial mass or decreased mitochondrial membrane integrity.

### Primary Human CGD Phagocytes Have Elevated Levels of Mitochondria-Derived ROS

**Figure 5:**
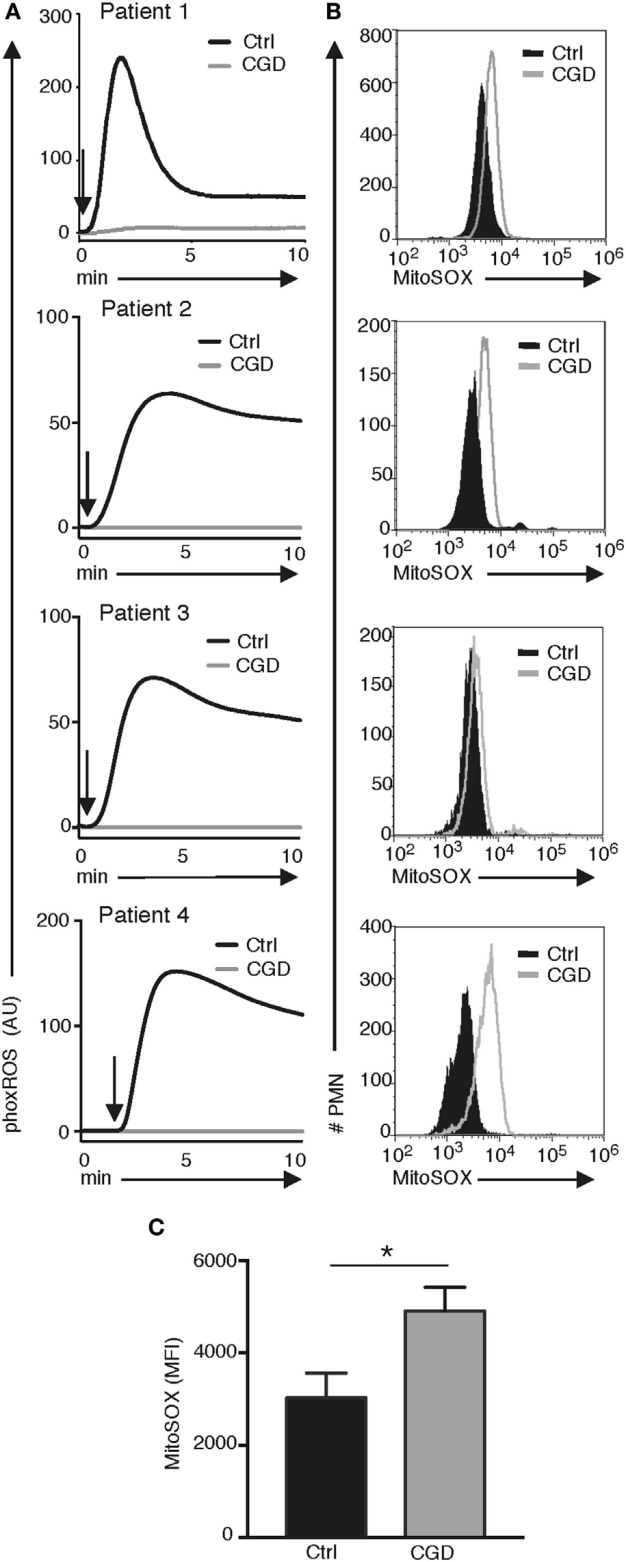
phoxROS and mt ROS production by primary (CGD) phagocytes. Primary blood cells from four patients with CGD (gray lines) and four healthy individuals (black lines) were analyzed for **(A)** phoxROS production in response to (PMA; addition of stimuli is indicated by arrows) as measured by (CL) and **(B)** MitoSOX fluorescence in resting, non-activated PMN; histograms are representative from triplicate stainings for patients 2–4 and a single measurement for patient 1. In **(A)**, isolated PMNs were used for patients 2–4 and their respective controls and complete leukocyte preparations for patient #1 and its control donor. **(C)** Bargraph shows the (MFI) of MitoSOX fluorescence + SEM for all CGD patient and (*n* = 4) and controls (*n* = 4).

Based on our observation that mtROS is elevated in X-PLB compared to PLB phagocytes, MitoSOX fluorescence was quantified in blood cells from four individuals with CGD. We first confirmed that phagocytes from these individuals were unable to generate NADPH-oxidase-derived phoxROS as measured by CL (Figure 5A). Mitochondrial ROS, as detected by MitoSOX staining, was of significantly higher intensity in each of these patients’ phagocytes compared to phagocytes obtained from healthy individuals (Figure 5B showing flow cytometry histograms for each patient and Figure 5C showing the average MitoSOX staining for the patients versus the healthy individuals). These results are in agreement with the observation in the cell line that a deficiency in phoxROS is sufficient to cause an increase in intracellular ROS, originating from the mitochondria, as demonstrated by the increase in MitoSOX staining (Figure 5C).

### Elevated Levels of Mitochondria-Derived ROS Promote MAPK Signaling and Cytokine Production

**Figure 6:**
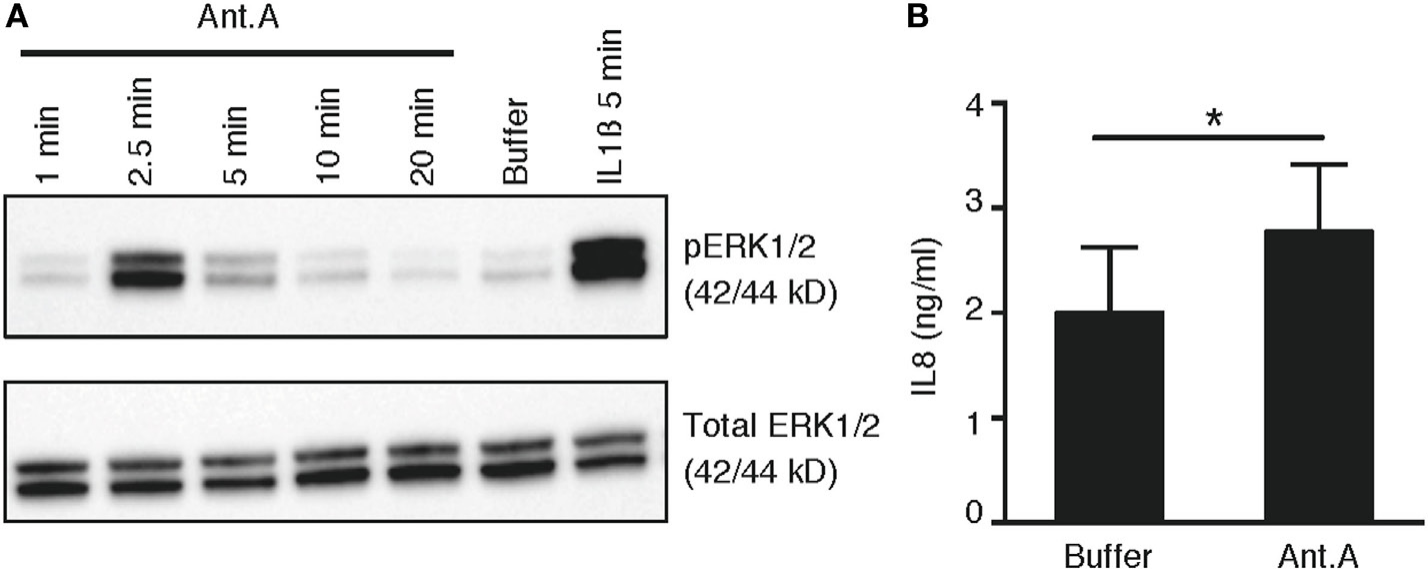
mtROS-induced phosphorylation of ERK1/2 and production of IL8. **(A)** Representative immunoblots of phosphorylated (top panel) and total (bottom panel) ERK1/2 in primary PMN (*n* = 3; buffycoats from healthy donors) following treatment with Antimycin A (Ant.A; 20 µM), buffer (KRG) or IL1β (100 ng/mL) for indicated time points. **(B)** Bar graph show the mean concentration + SEM of IL8 protein in lysates from PLB monocytes (*n* = 4) after incubation of cells in the absence (buffer) or presence of Antimycin A (20 µM) for 6 h (*n* = 4).

To provide mechanistic evidence to link our observed correlation between the elevated production of both pro-inflammatory cytokines and mtROS in phoxROS-deficient cell lines and primary phagocytes, we investigated the ability of Antimycin A, a specific inducer of mtROS (Figure 3A; Figure S2 in Supplementary Material), to directly induce intracellular signaling *via* phosphorylation of ERK1/2, and subsequent pro-inflammatory cytokine production (IL8). Our data demonstrate that Antimycin A rapidly enhanced phosphorylation of ERK1/2 (Figure 6A) and also induced production of IL8 (Figure 6B). Thus, elevated mtROS induces rapid activation of MAPK signaling *via* ERK1/2 that likely contributes to downstream transcription and secretion of cytokines, demonstrated as increased production of IL8 after treatment of cells with Antimycin A. These data are in agreement with our prior work demonstrating that elevated pro-inflammatory cytokine production by CGD phagocytes is dependent on constitutive nuclear translocation of NFκB and a functional ERK1/2 pathway (6, 7).

## Discussion

This study attempts to reconcile the observations that ROS are required for inflammatory signaling yet murine and human CGD phagocytes, which are devoid of the main source of ROS, have elevated inflammatory signaling and cytokine production (5–9). We demonstrate that phoxROS-deficient phagocytes (cell line as well as primary CGD cells) have higher levels of ROS derived from mitochondria (mtROS). Coincident with this, mitochondrial and cytosolic antioxidants (SOD2 and TXN) as well as reduced surface thiols are elevated as compared to phoxROS-competent phagocytes. Previous studies have implicated mtROS in a variety of stimuli-induced pro-inflammatory signaling events (19–21). To our knowledge, our data are the first to suggest that basal levels (i.e., in the absence of external stimuli) of cytokine production is also dictated by mtROS and operates at least in part by rapid engagement of the ERK1/2 MAPK pathway. In this context, there may be a role for mtROS in persistent signaling and cytokine production associated with sterile inflammatory diseases. Our data also support the notion that CGD in many ways can be regarded as an autoinflammatory disease, e.g., abnormally increased inflammation, driven by dysregulation of molecules and cells of the innate immune system (33).

Our data with markedly increased levels of mtROS in CGD cells are not in agreement with a previous publication by Fernandez-Boyanapalli et al. that reported partial reversal of ROS production in CGD cells upon treatment with the PPARγ agonist pioglitazone (34). In this paper, it is shown that CGD cells display significantly *decreased* mtROS, as compared to wild-type phagocytes. However, this paper measured *induced* mtROS production (i.e., cells stimulated with PMA), whereas our focus is on resting cells and basal levels of mtROS. When we stimulate mtROS production without affecting phoxROS, using Antimycin A, we find that the induced mtROS also stimulates ERK1/2 phosphorylation and IL8 production. The Antimycin A-induced IL8 production (Figure 6B) was seen after only 6 h; longer incubation with Antimycin A was associated with cytotoxicity and in this short time we were unable to detect additional cytokines (such as TNF and IL6).

The elevated mtROS we observed in both primary cells and the cell line was concurrent with an overexpression (compared to control cells) of the mitochondria-specific antioxidant, SOD2. This indicates that in phoxROS-deficient phagocytes, mitochondrial oxidants are increased to a level that warrants an appropriate cellular (antioxidant) response aiming to restore redox balance. It seems, however, that this antioxidant response (including a 2.5-fold increase in *SOD2* expression alone) fails to sufficiently neutralize the surplus of mitochondrial oxidants in phoxROS-deficient phagocytes to levels of mtROS observed in control cells. It also suggests that our data may underestimate the differences in mtROS production between control and phoxROS-deficient phagocytes given that MitoSOX can only detect the fraction of mtROS that are not effectively neutralized by (elevated) endogenous antioxidants.

Like *SOD2, SOD1* is also constitutively elevated in primary CGD phagocytes (gene expression data not shown). The overexpression of *SOD2* and *SOD1* suggests that superoxide is released (and transformed into hydrogen peroxide) to both the intermembrane space and the mitochondrial matrix. Within the intermembrane space, the pool of superoxide and peroxide can be enhanced by the action of p66SHC (SHC1) and Mia40 (CHCHD4), respectively. However, we find no evidence that the genes encoding these proteins are expressed to different degrees in primary CGD and control phagocytes. This was also true for genes encoding peroxiredoxin II, and peroxiredoxin III that neutralize hydrogen peroxide to water in the intermembrane space and mitochondrial matrix, respectively. Taken together, these data suggest that excess superoxide anions are released from the electron transport chain into both the mitochondrial matrix and the intermembrane space of mitochondria in phoxROS-deficient (CGD) cells.

It has been appreciated for some time that the MAPK and NFκB signaling pathways, which promote the transcription of inflammatory cytokines, contain signaling molecules that are redox-sensitive (35, 36). The transcription factor NFκB is directly activated by oxidation (35) while activation of the MAPK pathway occurs by both oxidation-induced activation of MAP-kinases, and simultaneous inactivation of MAPK phosphatases (36). We previously demonstrated that the LPS-induced production of cytokines in primary CGD leukocytes was particularly sensitive to inhibition of the MAP-kinase ERK1/2 (6). Furthermore, in resting primary CGD cells, the NFκB inhibitor IκB was phosphorylated and degraded, and correspondingly, more of the p50 subunit of NFκB was localized in the nucleus (7). Herein, we demonstrate a causal link between elevation of mtROS levels, rapid phosphorylation of ERK1/2, and increased cytokine production.

Clearly, phoxROS may regulate immunity in a number of ways and, over the years, different mechanisms have been uncovered that could potentially help explain why CGD patients suffer from exaggerated inflammation. For example, CGD neutrophils live longer (37) and the lack of ROS from these cells results in an inability to inactivate pro-inflammatory mediators (38). Furthermore, CGD monocytes have been shown to exhibit defective autophagy which was linked to increased IL1β secretion (39). Also, there are findings that may have implications for the more autoimmune features of CGD pathology, e.g., that phoxROS regulate antigen processing and MHC-I cross-presentation in dendritic cells (40, 41), and also downregulate responses of autoreactive T cells (42). Our findings, that phoxROS-deficient phagocytes display enhanced levels of mtROS and are under oxidative stress, could help explain the puzzling paradox that ROS drives pro-inflammatory signaling yet CGD is a disorder associated with overwhelming inflammation. In addition, it has been argued that the hyper-inflammatory features of CGD are due to the presence of subclinical infections (and/or failure to properly degrade phagocytosed material) that continuously trigger inflammatory stimulation, making the inflammatory pathologies a direct consequence of suboptimal microbial killing. We believe that the fact that our data demonstrate increased basal cytokine production from the phoxROS-deficient cell line is clear evidence that phagocytes lacking a functional NADPH-oxidase are inherently autoinflammatory.

Our findings of elevated mtROS, oxidative stress, and cytokines in the absence of cellular stimulation are particularly pertinent for the understanding of the underlying mechanisms that drive chronic, sterile inflammatory disease in CGD. Our findings suggest that part of the pathology for CGD, a disease characterized by the lack of (phox)ROS, could in fact be due to an over-abundance of (mt)ROS. The data presented in this study are also in line with higher baseline levels of mtROS in neutrophils and monocytes obtained from patients with lupus and other inflammatory diseases (43). This implies that novel anti-inflammatory agents that target mitochondria or the cellular redox network could potentially be used to calm sterile inflammatory disorders that arise in CGD and other inflammatory and metabolic disorders that are sensitive to changes in cellular redox state.

## Ethics Statement

The study was approved by the University of British Columbia Clinical Research Ethics Board protocol with written informed consent obtained from the participants. Sampling of the Swedish patients was approved by the Regional Ethical Board of Gothenburg, Sweden with written informed consent obtained, or carried out as part of a clinical exploration to further understand the pathogenesis of the patients’ diseases. Written consent is not required by Swedish laws for procedures considered part of the clinical investigation of patients, but it is required that all relevant information regarding a patient’s investigations, clinical findings, procedures, etc., are documented in the patient’s file. Verbal informed consent was documented in the patient’s file as stipulated by The National Board of Health and Welfare (SOSFS 2008:14). This study also includes peripheral blood samples taken from healthy donors (ethics permit DNR: S010-03) and from buffy coats obtained from the blood bank at the Sahlgrenska University Hospital, Gothenburg, Sweden. According to the Swedish legislation section code 4§ 3p SFS 2003:460, no ethical approval was needed since the buffy coats were provided anonymously.

## Author Contributions

JB, KB, MS, DS, AF, CD, and AK designed the experiments. MS, KB, KC, HB, and VO performed the experiments. AF and DS recruited the patients. MS, KB, and JB wrote the paper and all the authors commented on the paper and approved the final version.

## Conflict of Interest Statement

The authors declare that the research was conducted in the absence of any commercial or financial relationships that could be construed as a potential conflict of interest.

## Abbreviations

ALM-633, Alexa Fluor^®^ 633 C5 maleimide; CBA, cytokine bead array; CGD, chronic granulomatous disease; CL, chemiluminescence; DCFDA, 2′,7′-dichlorofluorescin diacetate; DMSO, dimethyl sulfoxide; FCCP, carbonyl cyanide 4-(trifluoromethoxy) phenylhydrazone; FCS, fetal calf serum; HRP, horseradish peroxidase; KRG, Krebs–Ringer phosphate buffer; MFI, mean fluorescence intensity; mtROS, mitochondria-derived ROS; phoxROS, NADPH-oxidase-derived ROS; PMA, phorbol myristate acetate; qPCR, quantitative real-time PCR; ROS, reactive oxygen species; SOD2, superoxide dismutase-2; TMRE, tetramethylrhodamine; TXN, thioredoxin.
