# Supplementary material for: Elevated Mitochondrial Reactive Oxygen Species and Cellular Redox Imbalance in Human NADPH-Oxidase-Deficient Phagocytes
Source: Front Immunol. 2017 Dec 21;8:1828. doi: 10.3389/fimmu.2017.01828 (PMC5744066; doi:10.3389/fimmu.2017.01828)
Supplement: Supplementary file 1 [file Image_1.PDF]

Figure S1, Sundqvist *et al*

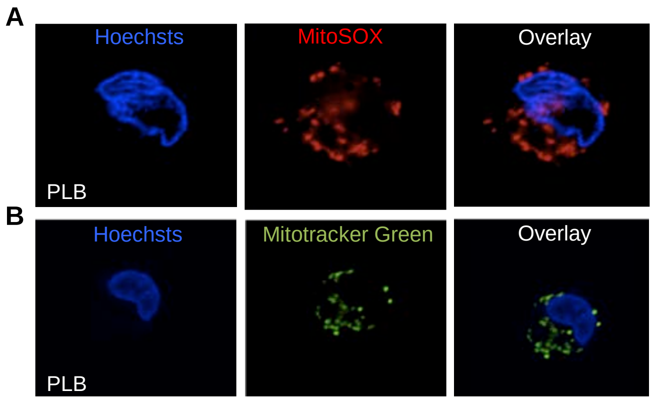

Figure S1. **Visualization of mitochondrial probes**.
PLB cells were matured to monocytes on glass-bottom, 24-well plates (MatTek Corp.) and then stained with Hoechst 33342 (3.5 µg/mL, Invitrogen, blue, left panels) and (**A**) MitoSOX™ Red (2.5 µM, red, middle panel, n = 3 independent experiments) or (**B**) Mitotracker Green FM (20 nM, green, middle panel, n = 2 independent experiments) at 37° C for a maximum of 30 min. Cells were then washed and visualized using a LSM 700 confocal microscope through a X63 apochromat objective with 2 times averaging.
