# Supplementary material for: Elevated Mitochondrial Reactive Oxygen Species and Cellular Redox Imbalance in Human NADPH-Oxidase-Deficient Phagocytes
Source: Front Immunol. 2017 Dec 21;8:1828. doi: 10.3389/fimmu.2017.01828 (PMC5744066; doi:10.3389/fimmu.2017.01828)
Supplement: Supplementary file 2 [file Image_2.PDF]

Figure S2, Sundqvist *et al*

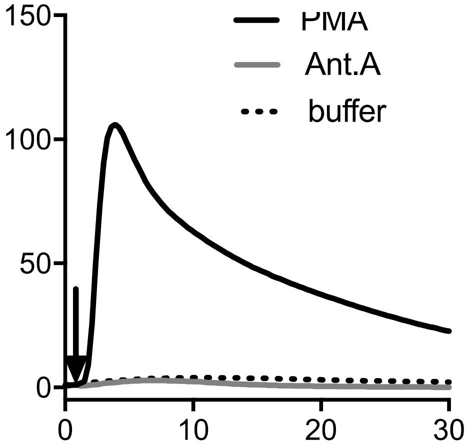

Figure S2. Antimycin A does not induce phoxROS production. Representative trace of phoxROS production (y-axis, Mega counts per minute, Mcpm) by primary buffycoat PMN from healthy donors (n = 3) over time (x-axis, min) following stimulation (indicated by the arrow) with PMA (50 nM, black line), Antamycin A (Ant.A, 20 µM, grey line) or buffer (KRG, black dotted line).
