# Supplementary material for: Elevated Mitochondrial Reactive Oxygen Species and Cellular Redox Imbalance in Human NADPH-Oxidase-Deficient Phagocytes
Source: Front Immunol. 2017 Dec 21;8:1828. doi: 10.3389/fimmu.2017.01828 (PMC5744066; doi:10.3389/fimmu.2017.01828)
Supplement: Supplementary file 3 [file Image_3.PDF]

Figure S3, Sundqvist *et al*

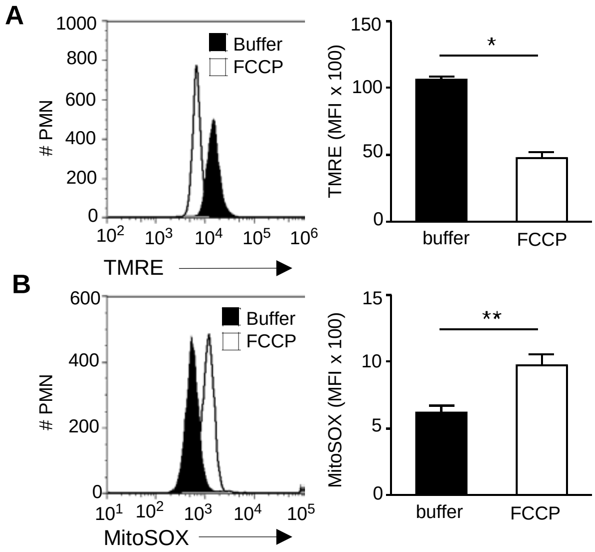

Figure S3. Effect of FCCP on TMRE and MitoSOX staining in primary neutrophils.

Primary buffycoat PMN (5 x 10^5) from healthy donors were incubated without (buffer, black) and with (white) FCCP (20 µM) for 10 min before staining with (**A**) TMRE (400 nM) or (**B**) MitoSOX Red (5 µM) for 30 min at 37°C, 5% CO_2. A minimum of 10 000 gated (FSC vs SSC) PMN were collected on an Accuri C6 flow cytometer and analyzed with FlowJo software. Left panel show representative flow cytometry histograms and right panels show the average MFI + SEM from three independent experiments.
